# Auditory-Motor Learning during Speech Production in 9-11-Year-Old Children

**DOI:** 10.1371/journal.pone.0012975

**Published:** 2010-09-24

**Authors:** Douglas M. Shiller, Vincent L. Gracco, Susan Rvachew

**Affiliations:** 1 École d'orthophonie et d'audiologie, Université de Montréal, Montreal, Quebec, Canada; 2 CHU Sainte-Justine Research Center, Montreal, Quebec, Canada; 3 Centre for Research on Language, Mind and Brain, McGill University, Montreal, Quebec, Canada; 4 School of Communication Sciences and Disorders, McGill University, Montreal, Quebec, Canada; 5 Haskins Laboratories, New Haven, Connecticut, United States of America; CNRS, France

## Abstract

**Background:**

Hearing ability is essential for normal speech development, however the precise mechanisms linking auditory input and the improvement of speaking ability remain poorly understood. Auditory feedback during speech production is believed to play a critical role by providing the nervous system with information about speech outcomes that is used to learn and subsequently fine-tune speech motor output. Surprisingly, few studies have directly investigated such auditory-motor learning in the speech production of typically developing children.

**Methodology/Principal Findings:**

In the present study, we manipulated auditory feedback during speech production in a group of 9–11-year old children, as well as in adults. Following a period of speech practice under conditions of altered auditory feedback, compensatory changes in speech production and perception were examined. Consistent with prior studies, the adults exhibited compensatory changes in both their speech motor output and their perceptual representations of speech sound categories. The children exhibited compensatory changes in the motor domain, with a change in speech output that was similar in magnitude to that of the adults, however the children showed no reliable compensatory effect on their perceptual representations.

**Conclusions:**

The results indicate that 9–11-year-old children, whose speech motor and perceptual abilities are still not fully developed, are nonetheless capable of auditory-feedback-based sensorimotor adaptation, supporting a role for such learning processes in speech motor development. Auditory feedback may play a more limited role, however, in the fine-tuning of children's perceptual representations of speech sound categories.

## Introduction

The first several years of a child's life are characterized by dramatic improvements in speaking ability. At one month of age, infants are able to produce only a small range of vowel-like “cooing” sounds using crude, undifferentiated movements of the oral articulatory system. By age 4, children are not only able to produce a wide range of phonetically distinctive consonant and vowel sounds, but rapidly combine them into complex word forms yielding speech output that is fully intelligible [1], [2]. Speech development, however, does not end with the establishment of intelligible speech production. Subsequent improvement of speech motor output continues through adolescence, characterized by a gradual reduction in variability in the timing of speech production [3], [4], [5], [6], [7], [8], [9], articulatory kinematic patterns [10], [11], [12], [13], [14], [15], and consequent acoustic spectral measures [6], [16], [17]. The gradual reduction in variability is accompanied by an increase in speaking rate [6], [15], [18] and the eventual achievement of more adult-like acoustic and kinematic parameter values (e.g., mean vowel formant frequencies and movement amplitudes; [6], [15], [19], [20], [21]).

While such age-related changes in speech ability have been well documented, our understanding of the mechanisms driving these changes remains incomplete. Speech motor development has been linked to changes that are occurring in parallel in the domains of anatomical [21], [22], perceptual [23], motor [24] and linguistic [11], [25] development. Speech motor learning, in particular learning based on auditory-feedback, is also presumed to play a major role in speech development (e.g., [26], [27]), however surprisingly few studies have directly examined children's capacity to use auditory feedback in order to adjust their control of speech output.

In the present study, we investigated children's capacity to adapt their speech production to an experimental manipulation of auditory feedback. Similar studies of *sensorimotor adaptation* (SA) have been explored previously in adults, involving changes in auditory feedback related to a number of acoustic spectral parameters including vowel formant frequencies [28], [29], [30], [31], [32], fundamental frequency [33], [34], [35], and fricative first spectral moment [36]. These studies have all reported adaptive changes in speech output that counteract the effects of the auditory feedback manipulations following a period of intensive speech practice. In current models, the process of sensorimotor adaptation is presumed to result from plasticity in neural representations of sensory-motor relationships (internal models) that are used in the coordination of voluntary movements [27], [33], [37]. In addition, the process of sensorimotor adaptation appears to depend upon accurate speech perceptual abilities, as demonstrated in a recent study by Villacorta et al., [32] linking SA performance with auditory acuity in adult talkers.

We have recently extended these findings by demonstrating, in addition to speech motor compensation following a manipulation of the subject's auditory feedback, an adaptive change in the *perception* of the manipulated sound category [36]. Specifically, following a feedback shift involving a reduction in/s/centroid frequency (in the direction of the category/∫/, or “sh”), the location of subjects'/s- ∫/category boundaries was observed to similarly shift toward a lower centroid frequency. A control group who passively listened to a matched sequence of frequency altered/s/- stimuli did not show such a perceptual adaptation effect, indicating that the perceptual changes were linked to the auditory-motor adaptation. The finding supports the idea that, in adults, SA to altered auditory feedback is not limited to the motor domain, but rather involves complementary changes in both sensory and motor processes that act to maintain the achievement of speech goals [36].

Little is known about the capacity for SA in children, for whom sensory and motor processes related to speech production are not yet fully developed. A number of investigations of SA have been carried out in children involving mechanical perturbations to the oral articulators during speech production. These manipulations, which have included jaw fixation using a bite-block [38], [39], [40], [41], [42], [43] and lip fixation using a tube held between the lips during vowel production [44] simultaneously alter orosensory and auditory feedback while limiting the degrees of freedom of the articulators during speech production. As such, they are complex manipulations that require significant changes in the coordination of articulator motion in order to compensate. Studies employing these methodologies have yielded mixed results, with some indicating a limited capacity of children to adapt [40], [41], [44], and others demonstrating comparable degrees of speech adaptation between children and adults [38], [39], [42], [43]. Because of the multisensory nature of these manipulations, it is difficult to separate the roles of auditory and orosensory feedback in the resulting motor adaptive effects.

In a recent study by Walsh et al. [45], short-term plasticity in the control of lip/jaw movement was examined during speech production in a group of 9–10-year-old children. When producing a novel non-word phoneme sequence, the children initially exhibited a greater degree of kinematic variability in addition to longer overall movement durations relative to a group of adult controls. Following repetitions of the target sequence, the children showed a reduction in movement variability and duration (i.e., a practice effect), while adult performance (which was consistently better than that of the children) showed little improvement. The authors suggested that the short-term practice effect observed in the children may have resulted from sensory-feedback based adjustments in order to achieve a desired auditory goal, though without a direct manipulation of sensory feedback it was not possible to confirm this hypothesis.

Despite the central role attributed to auditory feedback in current models of speech development, prior studies have not provided clear evidence that typically developing children can readily use auditory input related to their own speech production to improve and maintain the quality of their speech output. In the present study, real-time acoustic signal processing was used to precisely manipulate a phonetic property of speech auditory feedback in a group of 9–11-year-old children without impacting other sensory modalities or interfering with articulator motion. The procedure thus allowed us to directly examine children's use of auditory feedback to maintain accurate control of segmental speech production, as well as to explore the possible use of auditory feedback in their fine-tuning of perceptual representations of speech sounds --- a role for auditory feedback that has not been previously explored in children. Given likely critical role for sensory-based learning processes in children's speech development, we predicted that the children would exhibit sensitivity to changes in auditory feedback that result in compensatory changes in speech motor output, as well as in their perceptual representation of the phoneme category. With respect to the relative *degree* of motor and perceptual adaptation effects between the two age groups, however, three possible outcomes may be hypothesized: 1) given that sensory-based adaptation depends upon accurate perceptual and motor processing, limitations in children's speech motor and perceptual ability might result in a *reduced* degree of auditory-feedback-based motor and perceptual learning for children in comparison to adult talkers; 2) the children's perceptual and motor abilities, while not yet fully developed, may nonetheless be sufficient to achieve adult-like learning performance; or 3) the children may exhibit *stronger* learning effects than adults, owing to their increased neural plasticity and/or less well established motor and perceptual representations.

## Methods

### Ethics Statement

All subjects (or, for minors, their parent/guardian) gave their written informed consent to participate in the study, which was performed with approval of the Institutional Review Board of the Faculty of Medicine at McGill University.

### Subjects

Two groups of subjects were tested: one consisting of 11 children (*C*-group, age 9 yrs, 5 months - 11 yrs, 3 months; 5 female and 6 male), and another consisting of 13 adults (*A*-group, age 23–30 years, 6 female and 7 male). All subjects were native speakers of North American English, with no reported history of speech or language disorder and no hearing impairment. For each subject, a pure-tone hearing screening carried out immediately prior to testing confirmed that hearing thresholds were below 20 dB HL at 250, 500, 1000, 2000, 4000 and 8000 Hz.

### Procedures

Subjects were seated in a sound attenuating testing room (Industrial Acoustics Company) and spoke into a condenser microphone (ME-66, Sennheiser, Germany) positioned 10 cm from the mouth. The microphone signal was amplified to line level, digitized at 16-bit/44.1 kHz using an analog-to-digital converter (Transit, M-Audio, Irwindale, CA), and then recorded on a PC using Matlab (v.7.4, Mathworks, Natick, MA) and the Data Acquisition Toolbox (v. 2.10, Mathworks, Natick, MA).

Productions were cued by a combined written and pictorial representation of the target word (e.g., a picture of soup combined with the text “soup”) on a 21-inch computer display at a distance of 1.5 meters. Each visual stimulus was presented for 3 seconds, followed by a 1 second period in which the display was blank. Subjects were instructed to produce the target word at a comfortable speaking rate immediately following the onset of the visual cue. Speaking volume was maintained at a consistent, comfortable level throughout the procedure by providing visual feedback to the subject during a brief practice period as well as throughout the course of testing. The feedback was in the form of a digital VU meter (PPM ME12, v. 1.41, Darkwood Designs) presented on the computer display and calibrated to register a value 4 on a scale of 0 to 7 when the subject's speech amplitude was 65 dB SPL, as measured at the microphone 10 cm from the mouth.

All subjects carried out the following sequence of tasks:

1. Acclimatization: Subjects read aloud a sequence of 90 words into a microphone while listening to their amplified, but otherwise unaltered, speech acoustic signal through headphones (SR-80, Grado Labs, Brooklyn, NY). The stimuli consisted of an equal proportion of words beginning with/s/and/∫/, drawn from a set of 20 items (10/s/-words and 10/∫/-words; see Table 1). The words had the form: consonant-vowel (CV) or consonant-vowel-consonant (CVC), containing a range of vowel sounds and the final unvoiced stop consonants/p/,/t/, or/k/.
2. Phoneme identification pre-test: Following the acclimatization period, subjects underwent the first of two phoneme identification tasks which involved listening to synthetic speech stimuli through headphones and assigning a phoneme label to each token by responding on a computer keyboard (see *Phoneme Identification Task* below for details).
3. Speech production pre-test: Subjects underwent an assessment of speech production involving the production of/s/followed by the three English vowels:/u/(“sue”),/i/(“see”) and/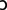/(“saw”) in order to introduce a degree of phonetic context-related variability into the/s/ productions. Each word was produced 10 times, in a fully randomized order. An additional 15 tokens of the word /∫u/ (“shoe”) were also included in the assessment in order to evaluate the baseline production contrast between /s/ and/∫/.
4. Speech practice: Subjects produced a random sequence of 120/s/-words drawn from the set of 10/s/-stimuli (Table 1). The first 10 trials were produced under unaltered feedback conditions, followed by the introduction of the acoustic perturbation (linearly ramped on over 10 trials), and then 100 trials under conditions of maximal acoustic perturbation (−3.0 semitones; see *Manipulation of Auditory Feedback* below for details).
5. Speech production post-test: A replication of the *speech production pre-test* (item 3 above), carried out under conditions of maximum auditory perturbation (−3 semitones). Compensatory changes in/s/were assessed as the difference in centroid frequency between this test and the speech production pre-test.
6. Phoneme identification post-test: Following the speech production post-test, subjects in both groups underwent a second phoneme identification procedure (same as Procedure 2 described above). Results of this post-test were compared with the pre-test in order to evaluate changes in perceptual representation of the/s- ∫/contrast following training.

**Table 1:** Speech stimuli.

/s/ stimuli	sue, see, saw, sack, sew, sip, sock, say, suck, soup
/∫/ stimuli	shoe, she, shop, shack, show, ship, shock, shake, shut, shoot,

### Phoneme Identification Task

The procedure for evaluating the perception of the/s- ∫/contrast involved the identification of synthetic fricatives that varied along an eight-step continuum from/s/to/∫/(for details about the stimuli, see [36], [46]). Individual stimuli were presented through headphones at a comfortable volume. Subjects identified each stimulus by pressing a key labeled “s” or “sh” on a computer keypad. Key order was counterbalanced between subjects, with half of the subjects in each group using the reverse key sequence. Each of the eight stimuli was presented 10 times in a fully randomized order. An additional 12 stimuli were added as practice trials at the beginning of each session, resulting in a total of 92 tokens presented per testing session.

### Manipulation of Auditory Feedback

Manipulation of/s/acoustics involved a change in the first spectral moment (or frequency *centroid*): a measure of central tendency in the spectral domain, computed as the amplitude-weighted mean of the frequency spectrum obtained by discrete Fourier transform. The fricative centroid is a stable, perceptually contrastive property of the sibilant fricatives/s/and/∫/[47], [48], and has been used to evaluate the accuracy of/s/production in a number of studies involving speech adaptation [36], [49], [50].

A commercial DSP (SPX-1000, Yamaha, Japan) was used to reduce the centroid frequency of the fricative/s/by 3 semitones (averaging -1222 Hz across subjects), resulting in an acoustic signal that was closer in centroid frequency to the fricative/∫/. Details about the DSP and its use in altering fricative spectral properties (including an empirical evaluation of the DSP's ability to manipulate/s/acoustics) can be found in Shiller et al. [36]. Because the processor remained active throughout each utterance, the frequency spectrum of the following vowel (including the fundamental frequency and all formants) was also shifted to the same degree, which had the effect of lowering the perceived pitch of the voice. Following the procedure described in Shiller et al. [36], the spectrally altered acoustic signal was amplified sufficiently in order to limit subjects' perception of their unaltered air/bone conducted speech signal (masking noise was not added due to its potential impact on the perceived noise spectrum of the fricatives). Sample audio files demonstrating the acoustic manipulation are provided as Supporting Information (Audio S1 and S2). The files are of a female adult talker producing the syllables/si/,/su/and/s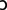/. Both the unmodified (Audio S1) and frequency-shifted output (Audio S2; as presented to the subject) are provided.

During the experiment, DSP settings were controlled using the PC and coordinated with the presentation of visual stimuli and audio recording using custom software routines written in Matlab (Mathworks, Natick, MA).

### Data Analysis – Acoustics

For each/s/- and/∫/-production during the speech production pretest and post-test, a 50 ms portion of the signal centered about the midpoint of the fricative was extracted using a custom program written in Matlab. The frequency centroid was computed for each extracted segment using the spectral moment function in PRAAT (v. 5.1.2, Boersma & Weenink, http://www.praat.org/). Baseline measures of/s/- and/∫/-production were obtained for the C and A groups by examining the productions of/s/- and/∫/-words during the speech production pretest. While the original intention was to compare/s/and/sh/solely within the context of the vowel/u/, /s/-productions were collapsed across the two back-vowel contexts (/su/and/sa/) for the purpose of the analysis as no reliable difference was observed in/s/-centroid values between these conditions for either group (C-group: *t*(10) = 0.37, *p* = 0.70; A-group: *t*(12) = 0.8, *p* = 0.43).

The change in fricative production following the SA procedure was evaluated on the basis of the speech production pre-test and post-test. Following Shiller et al. [36], mean/s/-centroids (averaged across all three vowel contexts) were obtained for each subject's pre- and post-test, and then a difference score was computed (post-test - pre-test) to determine the direction and magnitude of each subject's speech practice effect. Centroid values were averaged across the three vowel contexts because, as in Shiller et al. [36], it was confirmed that vowel context in the present study had no reliable impact on the magnitude of the/s/-motor adaptation effect in either the C-group (one-way repeated-measures ANOVA: *F*(2,20) = 1.53, *p* = 0.24) or A group (*F*(2,24) = 0.376, *p* = 0.69).

The reliability of the practice effects was evaluated using multiple *t*-tests (two-tailed, repeated measures for within-group comparisons, independent measures for between group comparisons), corrected for multiple comparisons (familywise *p*<.05) using Holm's sequential Bonferroni procedure.

### Data Analysis - Phoneme Identification Function

The/s- ∫/identification function was estimated for each subject from the set of response data obtained during the two phoneme identification tasks (pre- and post-test). The proportion of “s” responses was first computed for each of the eight stimuli (1.0 = 100% “s” response). These data were then linearly interpolated to an interval of 0.1 stimulus steps and a four-parameter logistic function (sigmoid) was fit to the resulting data points. The perceptual boundary between “s” and “sh” categories was defined as the point at which the proportion of “s” responses was 0.5. The slope of the identification function at the boundary provides an indication of the subject's difficulty in perceiving the phonetic contrast in the vicinity of the phoneme boundary (shallower slope  =  less consistent responses in the boundary region).

For each subject, sigmoid boundary estimates from the two assessments were converted to a difference score in order to determine the direction and magnitude of any practice effect. The reliability of perceptual adaptation effects within and between groups was carried out using *t*-tests, corrected for multiple comparisons (familywise *p*<.05) using Holm's sequential Bonferroni procedure.

## Results

### Baseline measures

#### Production of the/s-∫/contrast

Baseline measures of/s/and/∫/production were estimated on the basis of the speech production pretest. Mean/s/-centroid values for the children and adult groups were found to be similar, averaging 7645 Hz and 7724 Hz respectively, with no reliable difference observed between groups, *t*(22) = 0.27, *p* = .79. In contrast, the /∫/-centroid did show a reliable difference between groups, *t*(22) = 2.6, *p*<.05, averaging 5532 Hz for the children and 4997 Hz for the adults. The higher/∫/centroid produced by the children resulted in a reduced/s-∫/production contrast for that group (calculated as the difference between mean/s/and/∫/centroids), averaging 2113 Hz for children and 2726 Hz for the adults, *t*(22) = 2.50, *p*<.05.

The token-to-token variability of/s/and/∫/production was estimated for each subject by computing the standard deviation of centroid values for /s/ and /∫/. Overall, the variability of /s/ production was reliably greater for the children than for the adults (SD  = 681 Hz vs. 471 Hz respectively), *t*(22) = 3.71, *p*<.05. Similarly, /∫/-variability was found to be greater for the children than for the adults (SD  = 477 Hz vs. 302 Hz), *t*(22) = 3.54, *p*<.05.

**Figure 1:**
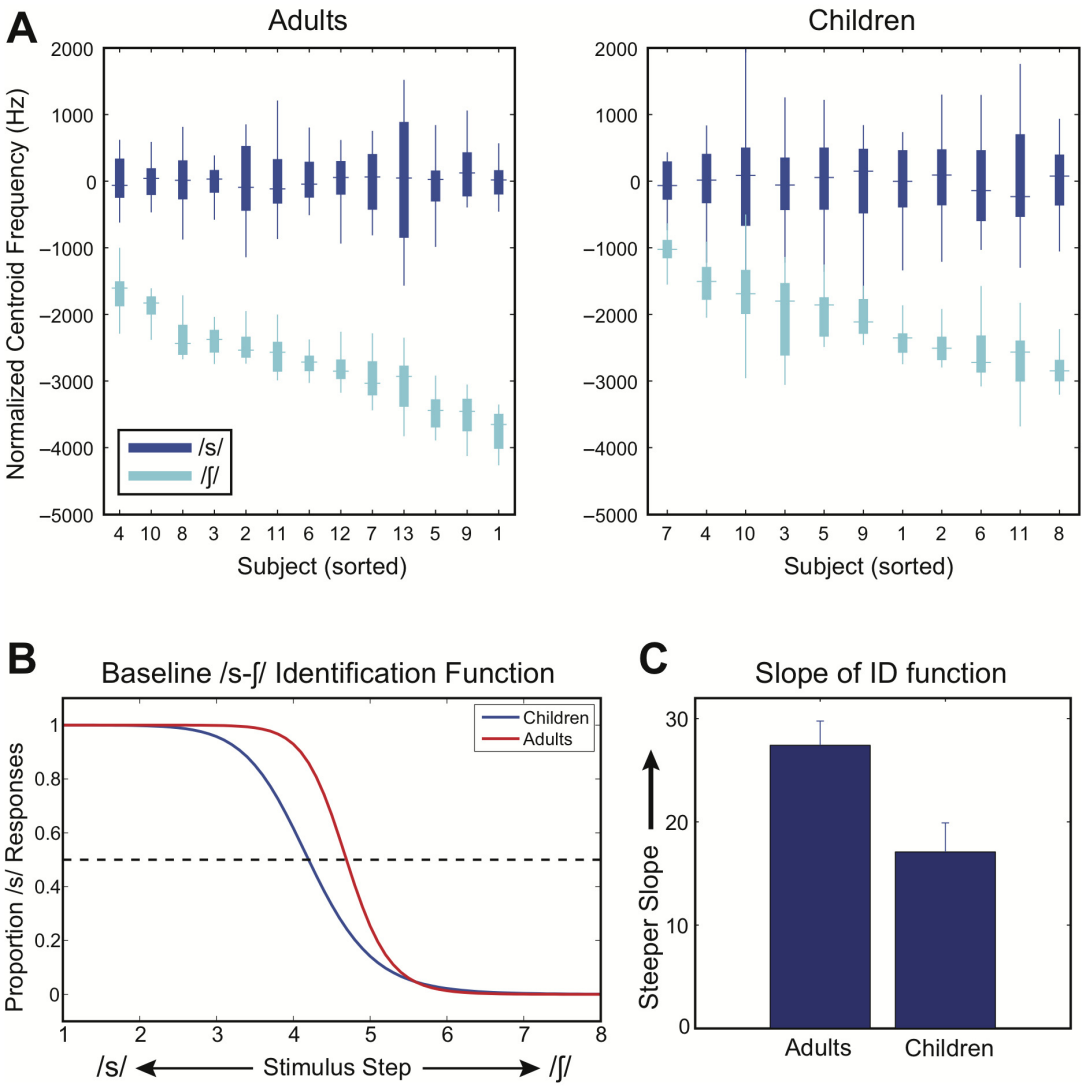
Baseline measures of /s/ and /∫/ production and perception. A. Boxplots of /s/ and /∫/ productions for individual subjects in each group. For visualization purposes, centroid values were normalized by subtracting the mean /s/-centroid frequency on an individual basis, and the subjects within each group were sorted on the basis of the magnitude of the production contrast. B. Mean /s-∫/ identification functions (sigmoid) for each group. C. Mean slope parameter values for the sigmoid-functions fit to each subject's pattern of responses in the phoneme identification task. Error bars show one standard error of the mean.

For several subjects in the children's group, the combination of greater trial-to-trial variability and reduced production contrast resulted in overlapping distributions of /s/ and /∫/ centroid frequencies. In contrast, no subjects in the adult group exhibited overlapping /s/ and /∫/ distributions. To illustrate the range of production contrasts and variability measures across subjects, boxplots of /s/ and /∫/ productions for all subjects are provided in Figure 1A. To better visualize the results, the centroid values were first normalized by subtracting the mean /s/-centroid frequency on an individual basis, and the subjects within each group were sorted on the basis of the magnitude of the production contrast.

#### Perception of the /s-∫/ contrast

The baseline perception of the /s-∫/ contrast was examined using the phoneme identification pretest, which was carried out immediately prior to the auditory feedback manipulation. Mean /s-∫/ identification functions for each group are plotted in Figure 1B, along with mean values for the slope parameters of the sigmoid function fit to each subject's pattern of responses in Figure 1C. Compared with the adult group, the children's group exhibited a more imprecise perceptual boundary between /s/ and /∫/ categories, as indicated by a smaller slope value relative to the adult group, *t*(22) = 2.83, *p*<.05. The location of the phoneme identification boundary (50% “s” responses) was also found to differ between groups, with the children's boundary lying reliably closer to the /s/-end of the /s-∫/ continuum, *t*(22) = 2.05, *p*<.05.

### Compensation for altered auditory feedback

#### Production of /s/

**Figure 2:**
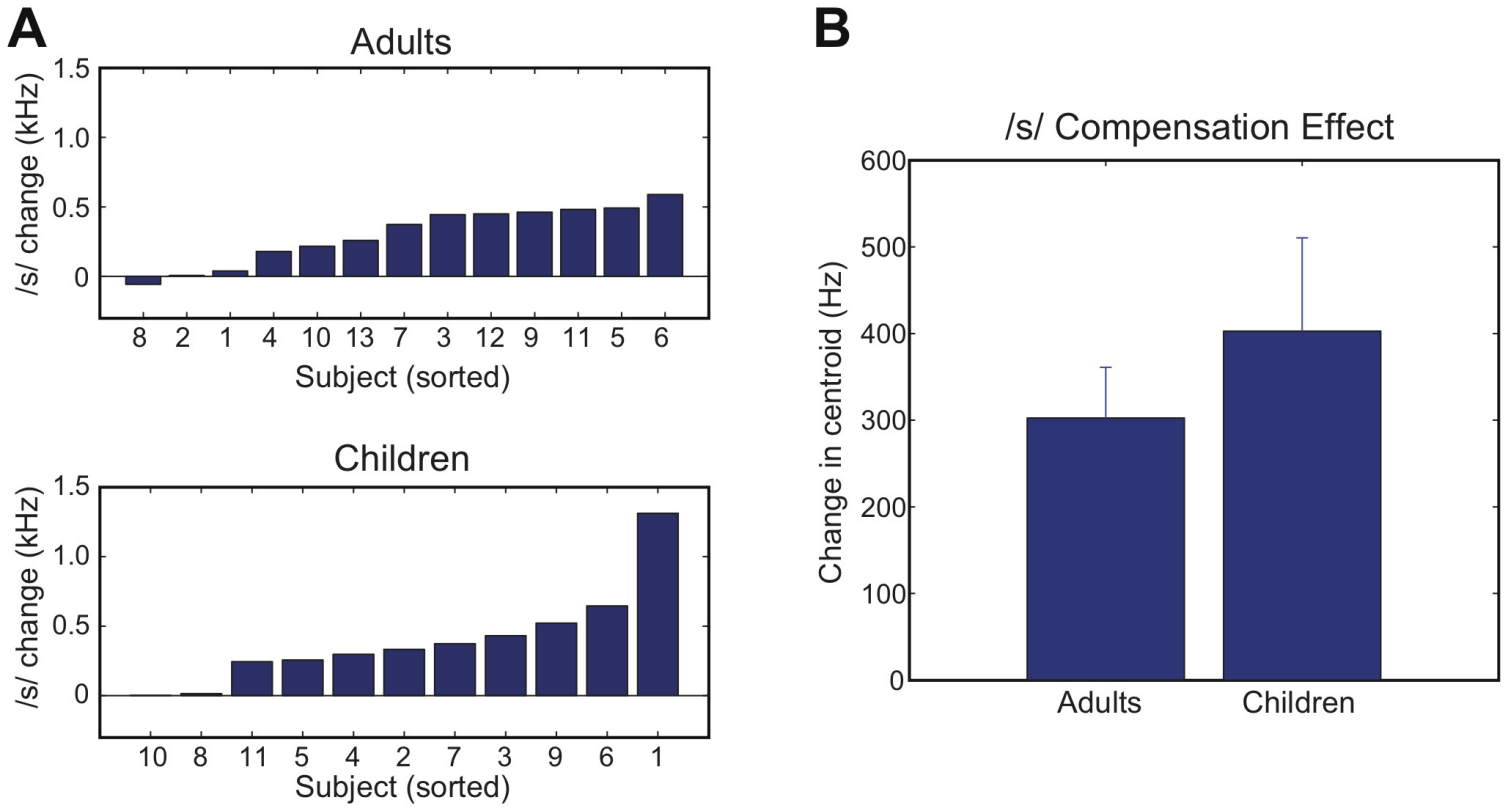
Changes in /s/-centroid frequency. A. Individual changes in centroid frequency within the adult (top panel) and child (bottom panel) groups. Changes are computed as the difference between the baseline phase and the end of the speech practice phase under conditions of altered auditory feedback. For visualization purposes, subjects are sorted on the basis of effect size. B. Group-mean change in /s/-centroid following speech practice under conditions of altered auditory feedback. Error bars show one standard error of the mean.

Because of the overall similarity in /s/-centroid frequency produced by the two groups, the three-semitone shift in /s/-centroid frequency yielded similar magnitudes of acoustic perturbation in both groups: averaging −1216 Hz for the children's group and −1229 Hz for the adult group. Following the period of speech practice under conditions of altered auditory feedback, a compensatory change in /s/-centroid frequency (i.e., an *increase* in centroid frequency that counteracted the auditory perturbation) was observed for a majority of subjects in both groups. Individual changes in /s/-centroid frequency are provided in Figure 2A. Examining the distribution of individual results, subjects in both groups are seen to exhibit a comparable range of motor compensation following training, with several individuals in each group showing near zero change and a majority of subjects increasing their /s/-centroid frequency in the range of 250–500 Hz. In the children's group, one individual (Subject 1) showed an unusually large increase of 1311 Hz.

Group means of /s/-centroid compensation are shown in Figure 2B. Within the adult group, the mean change in /s/-centroid value (post-test – pre-test, M = 303 Hz) was found to be reliable, *t*(12) = 5.18, *p*<.05. Within the children's group, the mean change of 403 Hz was also reliable, *t*(10) = 3.74, *p*<.05, and remained significant even with the omission of Subject 1 (M = 312 Hz), *t*(9) = 4.89, *p*<.05. The difference in /s/ compensation between groups (which is accounted for almost entirely by the large value observed for Subject 1 in the children's group) was not significant, *t*(22) = 0.85, *p* = .40.

#### Perception of /s-∫/ contrast

**Figure 3:**
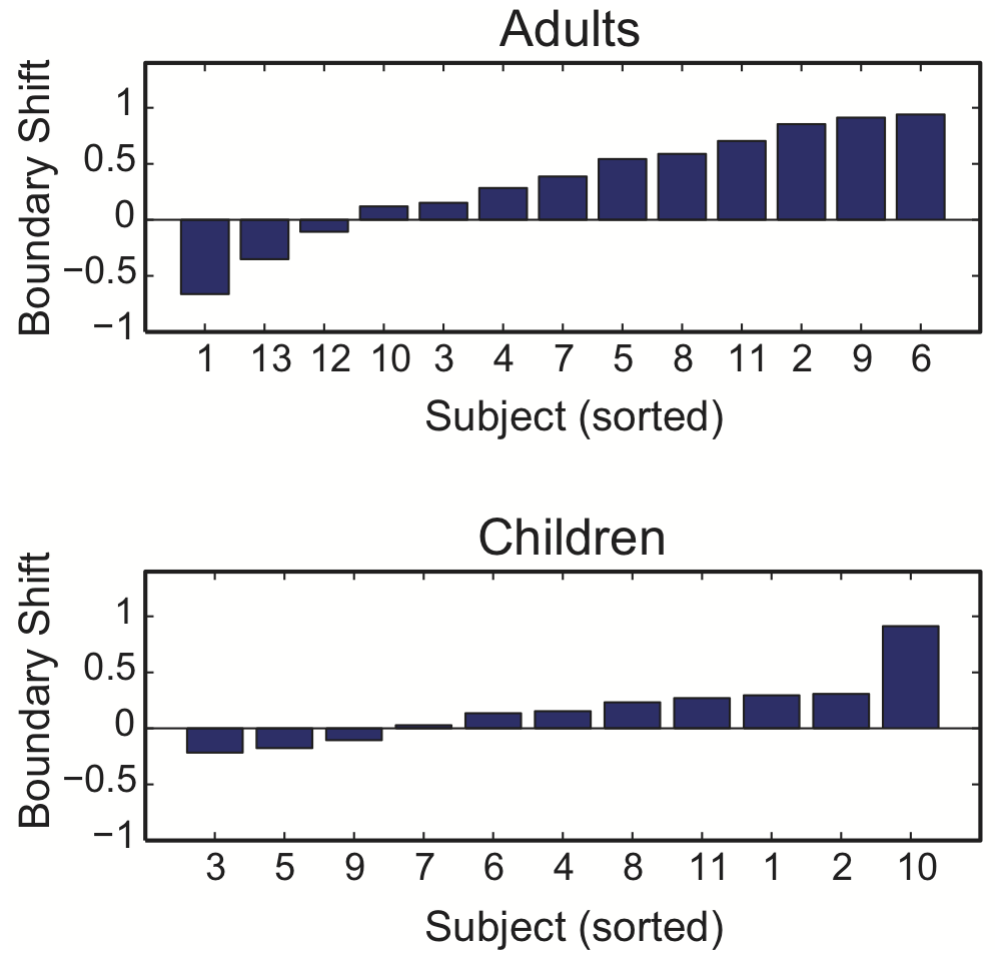
Shift in perceptual boundary for each subject. The figure shows the change in perceptual boundary following the period of speech practice (post-test – pretest), computed on the basis of individually estimated phoneme identification functions.

Perception of the /s-∫/ contrast was examined immediately prior to and following speech practice under conditions of altered auditory feedback. The change in location of the phoneme identification boundary was assessed on the basis of individually estimated phoneme identification functions. The computed boundary shift for each subject is shown in Figure 3. As in the case of /s/ production, considerable variability was observed between subjects in each group. In both groups, however, a majority of subjects exhibited a change in their perceptual boundary in the direction of the /∫/-end of the continuum.

**Figure 4:**
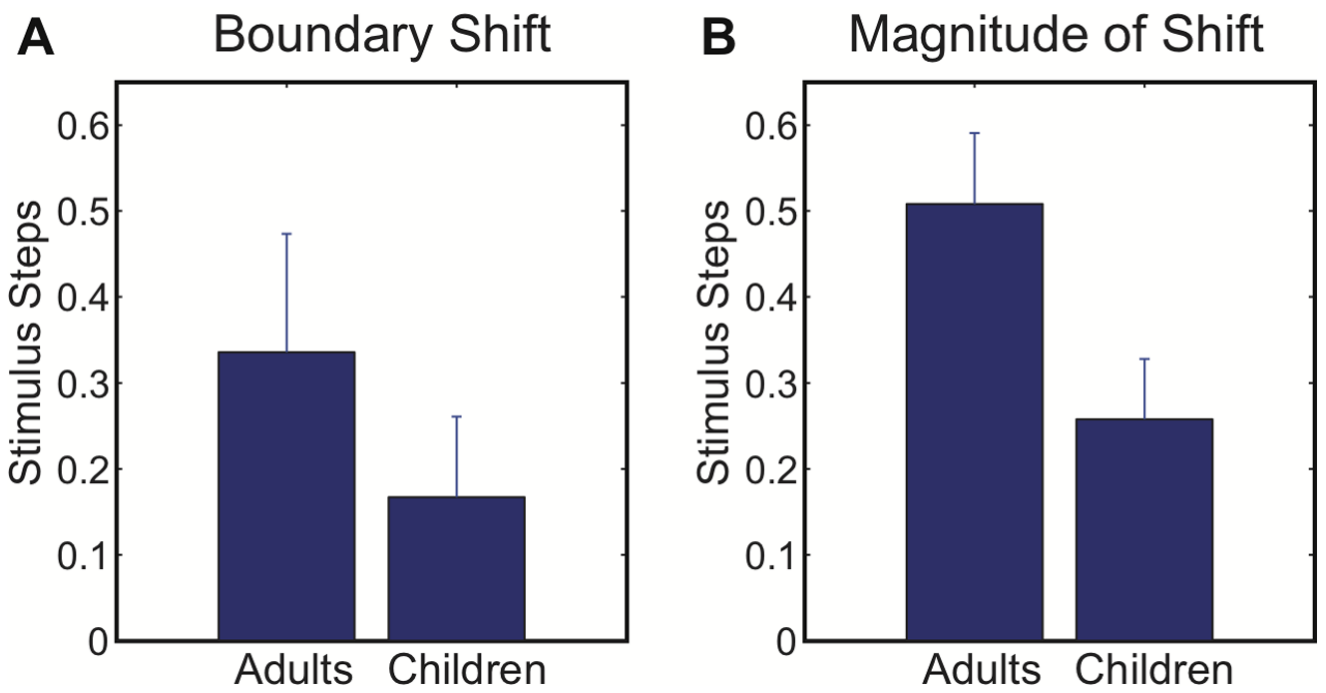
Change in perceptual boundary location. A. Mean change in perceptual boundary location for each group (post-test - pretest). B. Mean magnitude of the perceptual boundary effect for each group, irrespective of the direction of shift (averaging across absolute values of the perceptual boundary shift for each subject). Error bars show one standard error of the mean.

Mean values for the change in perceptual boundary location (post-test - pretest) are shown in Figure 4A. Subjects in the adult group exhibited a reliable change in boundary location in the direction of /∫/ (i.e., an adaptive shift) following the period of speech practice, *t*(12) = 2.44, *p*<.05. In contrast, the children's group exhibited a boundary shift that was on average smaller in magnitude and not reliably different from zero, *t*(10) = 1.8, *p* = .10. The between-group difference in boundary shift was not statistically reliable, *t*(22) = 0.98, *p* = 0.34, primarily owing to a large adaptive shift observed in Subject 10 in the children's group, and an unusually large shift in the *negative* direction (non-adaptive) for Subject 1 in the adult group (Figure 3). Omitting these two subjects (one from each group), the difference between adult and child groups was statistically significant, *t*(20) = 2.28, *p*<.05.

Note that the lack of observed perceptual adaptation in the children's group was associated with an overall smaller magnitude of perceptual boundary shift, and not from a greater proportion of subjects exhibiting a shift in the opposite direction (toward the /s/-end of the continuum). In both groups, a small number of subjects showed perceptual boundary shifts toward the /s/-end of the continuum (3 out of 13 in the adult group, and 3 out of 11 in the children's group). As can be seen in Figure 3, the children exhibited a relatively small magnitude of perceptual boundary shift following the feedback manipulation (with the exception of Subject 10), irrespective of whether the shift was in the positive (toward /∫/) or negative (toward /s/) direction. This difference can be quantified by computing the mean magnitude of the perceptual boundary effect for each group (averaging across absolute values of the perceptual boundary shift for each subject), as shown in Figure 4B. Although the absolute change in boundary location was significantly different from zero for both the adult group, *t*(12) = 6.14, *p*<.05, and the children's group, *t*(10) = 3.66, *p*<.05, the magnitude of the perceptual effect was found to be reliably greater for the adult group compared with the children, *t*(22) = 2.26, *p*<.05.

## Discussion

The children's baseline production and perception of the /s- ∫/ contrast were confirmed to be not fully adult-like, as evidenced by greater token-to-token variability, poorer production contrast between /s/ and / ∫/, and a less well defined perceptual boundary between /s/ and / ∫/. Following a period of speech practice under conditions of altered auditory feedback, sensorimotor adaptation was investigated in terms of changes in both the production and perception of /s/. Both the children and adults exhibited compensatory changes in speech motor output following the period of speech practice. Furthermore, the children exhibited a degree of speech motor compensation that was comparable to the adult group. This was somewhat surprising given the greater speech production variability and poorer production contrast between /s/ and / ∫/ production. Similar to previous results [36], the adult subjects showed a compensatory change in their perceptual boundary for the /s-∫/ contrast following the speech practice period. In contrast, the children exhibited a smaller change in their perceptual boundary that was not reliably different from zero. The results indicate that older children, whose speech motor and perceptual abilities are still not fully adult-like, are nonetheless capable of adaptive, auditory-feedback-based adjustments to their control of speech motor output. While it is difficult to draw strong inferences from a negative finding, the lack of observed perceptual adaptation in the children's group possibly suggests that auditory feedback may play a more limited role in the fine-tuning of perceptual representations of speech sounds in young talkers. This in turn suggests a continuing role for speech input from the environment (i.e., exogenous input) in children's developing speech perception ability (e.g., [51], [52]).

While the importance of auditory feedback in speech development has been recognized for some time (e.g., [53], [54]), earlier direct investigations of speech auditory feedback in children have been limited to studies of speech timing and amplitude. Studies in which speech auditory feedback was delayed by several hundred milliseconds have shown disruptive effects on the timing and fluency of children's speech production [55], [56], [57], [58], [59], in some cases, with children exhibiting stronger effects than adults (e.g., [58], [60]). Similarly, experimental manipulation of the perceived loudness of speech feedback (by adding noise or amplifying the acoustic signal), which typically results in a compensatory change in speech output volume in adults [61], has also been shown to elicit compensatory responses in children as young as 3-years of age [62], [63], [64]. These previous studies have been valuable in demonstrating that children attend to aspects of their own acoustic output during speech production. The present study extends these findings by providing a more direct examination of the role of auditory feedback in the achievement of phonetic targets. Specifically, we have demonstrated that children monitor spectral properties of their speech acoustic signal, making compensatory motor adjustments when necessary to maintain accuracy.

Previous studies of sensorimotor adaptation during speech production in adults have typically included an examination of learning *after-effects*: the persistence of any change in speech output following the sudden removal of the feedback manipulation after training [28], [29], [31], [32], [33], [34], [36]. Reliable after-effects have been found in all of these studies, indicating that the observed changes in speech output were the result of a modification of feed-forward motor plans (i.e., motor learning), as opposed to a change in motor output mediated by immediate sensory feedback (i.e., real-time feedback control). In the present study, which included tests of both motor and sensory adaptation, an examination of motor after-effects was not carried out in order to maintain a shorter testing time that would be tolerated by the child subjects. Given that reliable after-effects have been observed in nearly all prior studies of sensorimotor adaptation in speech production (including the manipulation of /s/-centroid frequency, as in Shiller et al., 2009), and given the likely impact of relatively long neural transmission times on the capacity of talkers to control articulatory movements using sensory feedback in real time [65], it is unlikely that the compensatory effects observed in the present study were entirely the result of direct auditory-feedback control. However, without an examination of learning after-effects, one cannot rule out the possibility that the children may have relied on direct sensory feedback control to a different degree than the adult talkers. Additionally, an examination of learning after-effects might reveal differences in the timing of de-adaptation that could possibly account for the difference in observed perceptual adaptation effects between the two groups. Further studies of sensorimotor adaptation in children will be required to address these questions.

In the present study, we explored a possible role of auditory feedback in children's fine-tuning of perceptual representations of speech sounds, in addition to changes in speech motor output. In Shiller et al. [36], using a protocol very similar to the one in the present study, adult talkers showed a change in the perceptual boundary that complemented the change in production. The finding suggested that the sensory and motor representations underlying speech production were tightly integrated, and that under conditions of altered auditory feedback, adaptive changes in both domains contributed to the maintenance of perceptual accuracy. In the present study, the adult group exhibited a perceptual adaptation effect similar to that observed in Shiller et al. [36], while the children's group exhibited no reliable adaptation in perceptual boundary. There are a number of possible explanations for this difference between the children and adults. The reduced perceptual adaptation effect in younger talkers may be directly related to the relative imprecision in their auditory representation of the sibilant contrast, as indicated by a shallower slope of the /s-∫/ identification function in the present study. As a result of this imprecision, the children may be less able to detect perceptual variability related to their own productions, and hence show less perceptual fine-tuning to accommodate such changes. The children's reduced perceptual adaptation effect may also be related to their increased *production* variability, which would result in greater variability in auditory feedback, and hence may limit the perceived reliability of the sensory input. Reduced sensory reliability has been shown to impact perceptual learning in the visual domain (see [66], for review), hence a similar principle may play a role in the children's processing of auditory feedback. Given that the children were able to successfully adapt their motor output to the perceived change in /s/ feedback, these explanations imply a dissociation between the auditory processing requirements for sensory and motor adaptation.

Yet another possible explanation for the lack of observed perceptual adaptation effect in children is that their auditory representations of speech sound categories are more resistant to short-term changes in auditory input than adults. While this may seem paradoxical, given that children are presumed to be endowed with plasticity in cognitive function that generally declines with age [67], there is some evidence that children as old as 10 years of age may be less susceptible than adults to short-term auditory learning effects such as *selective adaptation* (where repeated exposure to a speech sound biases later perception of that sound; [68], [69]).

The observation of successful, adult-like speech motor adaptation in 9–10-year-old children suggests that, while still immature, their capacity for sensorimotor processing is indeed sufficient to support precise feedback-based adjustments in speech motor control. Additional studies are required in order to determine whether younger children, with less accurate and more variable motor and perceptual abilities, exhibit greater difficulties adapting to short-term changes in auditory feedback.

## Supporting Information

Audio S1 Sample audio recording: Unmodified output. The file presents the unmodified acoustic signal of a female adult talker producing the syllables “sue”, “see” and “saw”.
(0.66 MB WAV)

Audio S2 Sample audio recording:Frequency-shifted output. The file presents the 3-semitone frequency-shifted audio signal (as presented to the subject in real-time) of the same female adult talker producing the syllables “sue”, “see” and “saw”.
(0.66 MB WAV)
